# Genome-wide analysis of ABA-responsive elements ABRE and CE3 reveals divergent patterns in Arabidopsis and rice

**DOI:** 10.1186/1471-2164-8-260

**Published:** 2007-08-01

**Authors:** Judith L Gómez-Porras, Diego Mauricio Riaño-Pachón, Ingo Dreyer, Jorge E Mayer, Bernd Mueller-Roeber

**Affiliations:** 1 University of Potsdam, Institute of Biochemistry and Biology, Karl-Liebknecht-Str. 24-25, Haus 20, D-14476 Potsdam-Golm, Germany; 2 Cooperative Research Group of the Max-Planck-Institute of Molecular Plant Physiology, Am Mühlenberg 1, D-14476 Potsdam-Golm, Germany; 3 Center for Applied Biosciences, University of Freiburg, Stefan-Meier-Str. 8, D-79104 Freiburg, Germany; 4 University of Bielefeld, Institute of Molecular Cell Physiology, Department of Biology, Universitätsstr. 25, D-33501 Germany

## Abstract

**Background:**

In plants, complex regulatory mechanisms are at the core of physiological and developmental processes. The phytohormone abscisic acid (ABA) is involved in the regulation of various such processes, including stomatal closure, seed and bud dormancy, and physiological responses to cold, drought and salinity stress. The underlying tissue or plant-wide control circuits often include combinatorial gene regulatory mechanisms and networks that we are only beginning to unravel with the help of new molecular tools. The increasing availability of genomic sequences and gene expression data enables us to dissect ABA regulatory mechanisms at the individual gene expression level. In this paper we used an *in-silico*-based approach directed towards genome-wide prediction and identification of specific features of ABA-responsive elements. In particular we analysed the genome-wide occurrence and positional arrangements of two well-described ABA-responsive *cis*-regulatory elements (CREs), ABRE and CE3, in thale cress (*Arabidopsis thaliana*) and rice (*Oryza sativa*).

**Results:**

Our results show that Arabidopsis and rice use the ABA-responsive elements ABRE and CE3 distinctively. Earlier reports for various monocots have identified CE3 as a coupling element (CE) associated with ABRE. Surprisingly, we found that while ABRE is equally abundant in both species, CE3 is practically absent in Arabidopsis. ABRE-ABRE pairs are common in both genomes, suggesting that these can form functional ABA-responsive complexes (ABRCs) in Arabidopsis and rice. Furthermore, we detected distinct combinations, orientation patterns and DNA strand preferences of ABRE and CE3 motifs in rice gene promoters.

**Conclusion:**

Our computational analyses revealed distinct recruitment patterns of ABA-responsive CREs in upstream sequences of Arabidopsis and rice. The apparent absence of CE3s in Arabidopsis suggests that another CE pairs with ABRE to establish a functional ABRC capable of interacting with transcription factors. Further studies will be needed to test whether the observed differences are extrapolatable to monocots and dicots in general, and to understand how they contribute to the fine-tuning of the hormonal response. The outcome of our investigation can now be used to direct future experimentation designed to further dissect the ABA-dependent regulatory networks.

## Background

Transcription is a complex process that requires a well-coordinated, multi-level regulatory machinery. Two key players of this delicate clockwork are transcription factors and their cognate *cis*-regulatory elements (CREs). Alone and in combination CREs contribute to the temporal and spatial diversity of expression patterns, and thereby provide, *inter alia*, the necessary control mechanisms involved in physiological responses to a myriad of internal and external triggers [1-3]. In eukaryotic organisms, the initiation of transcription by RNA polymerase II requires the assembly of the core transcriptional machinery with the core promoter of the target gene, generally located from 35 bases upstream to 35 bases downstream of the transcription start site [4]. Spatial constraints regarding the location of CREs in core promoters are known to some degree [4,5]. In addition to the basal transcription factors that are common to all transcribed genes and that form the core transcriptional machinery, numerous specific transcription factors bind to a whole gamut of usually short upstream DNA sequences, enhancing or repressing transcriptional activity in response to metabolic requirements, developmental stages or external stimuli. In contrast to the CREs in the core promoter, our knowledge of the positional and spatial arrangement constraints affecting regulatory elements further upstream is much more limited. Nonetheless, a frequency distribution approach has shown that distinct CRE enrichment patterns can be observed in the promoters of different eukaryotes [6]. In the present study we undertook the task to search for constraints in the arrangement of CREs in plant promoters, taking as a model two well-described elements – ABRE and CE3 – involved in transcriptional responses triggered by the phytohormone abscisic acid (ABA).

ABA plays a pivotal role in many physiological and developmental processes in plants. It is involved, among others, in the establishment of bud and seed dormancy, regulation of growth, leaf senescence and abscission, stomatal closure, and responses to various abiotic stresses, such as cold, drought and salinity [7-11]. This high degree of versatility is achieved through stringent regulatory control of ABA biosynthesis and hormonal cross-talk [12,13]. ABA regulates gene expression mainly at the level of transcription [10,14]. The molecular analysis of promoters of ABA-responsive genes has led to the identification of several motifs capable of conferring ABA responsiveness to a minimal promoter [3,11,15-21]. One such motif is the ABA-responsive element ABRE, which belongs to the so-called G-box family [3]. ABRE contains an ACGT core, a sequence known to be recognized by plant bZIP proteins [20,22-25]. It has been established that a single copy of ABRE is not sufficient for ABA-mediated induction of transcription, but multiple ABREs or the combination of an ABRE with a so-called coupling element (CE) can establish a minimal ABA-responsive complex (ABRC), and thereby confer ABA responsiveness to a minimal promoter [3,15,18-20]. Three coupling elements have been described, CE1, CE3 and DRE (dehydration responsive element), but as indicated above, an ABRE pair can also function as an ABRC, with the second ABRE playing the role of a coupling element [15]. The use of synthetic promoters has pointed to constraints on the orientation and spacing of ABRE and the CEs [18], albeit results from transient expression experiments may not fully reflect *in planta *distance and orientation requirements of ABA-responsive genes.

In the study presented here, we followed an *in-silico *approach to investigate genome-wide spatial constraints, such as strand preference, gap length between motifs, and distance to the translational initiation codon, affecting the occurrence of putative CREs in the genomes of *Arabidopsis thaliana *and *Oryza sativa*. As a model we used two well known CREs: ABRE and CE3. Our data revealed differences between the two genomes that point to evolutionary divergent patterns of ABA-responsive regulatory networks.

## Results

### Screening strategy

**Figure 1:**
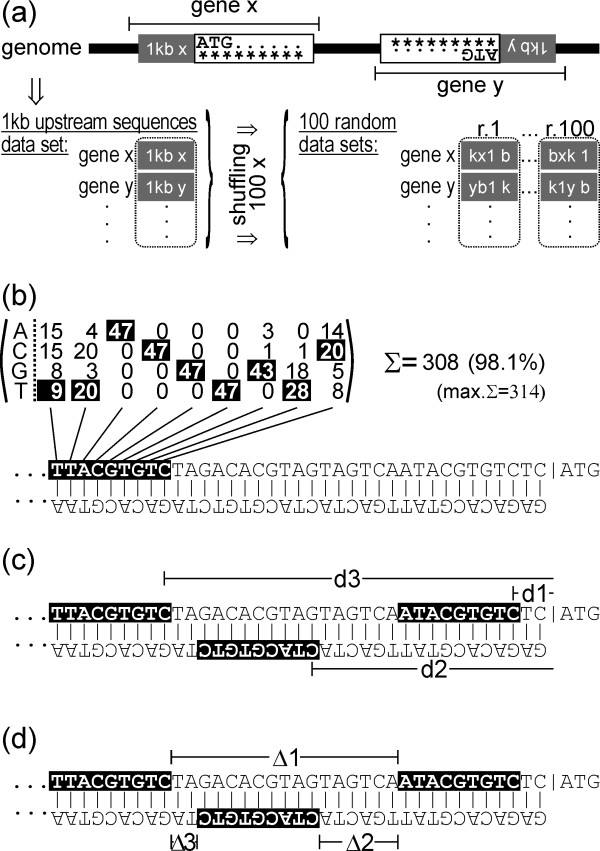
**Schematic outline of the data analysis strategy**. (a) Extraction of promoter sequences and generation of random data sets. For each annotated protein or peptide-coding gene the translation initiation codon (ATG) was located and a 1-kb upstream sequence was extracted. The genomic data set thus obtained contained 26,140 entries for *Arabidopsis thaliana *and 49,472 entries for *Oryza sativa*. For each species 100 independent randomised data sets were generated by reshuffling the nucleotide sequence of each entry. (b) *In-silico *screening using a CRE-specific frequency matrix. A positive hit was recorded if the matrix score reached a 95% cut-off threshold. (c) Determination of the distance of an element to the ATG. In the illustrated example d1 and d3 are the distances to the ATG for the elements localized on the [+] strand; d2 is the distance to the ATG for the element localized on the [-] strand. (d) Gap length between elements. In the example above Δ1 is the gap between two *cis*-oriented CREs (in this case [+/+]), and Δ2 and Δ3 are gaps between two *trans*-oriented CREs [-/+] and [+/-], respectively.

We searched for ABREs and CE3s in the promoter regions of protein-coding genes from *A. thaliana *and *O. sativa *using the genomic sequences of Arabidopsis (genome release 5.0, January 2004) and rice (release 4.0, January 2006) downloaded from The Institute for Genomic Research (TIGR) [26]. Due to scarce information on 5' untranslated regions (5'UTRs) for genes in the two genomes we opted for the use of 1-kb upstream sequences of predicted or verified translational start codons (ATG) instead. Beside the practical aspect, this approach also takes into account that a number of CREs are located within the 5'UTR [27]. A rice 1-kb upstream sequence database was obtained from TIGR. For Arabidopsis we extracted the corresponding regions from the full-genome sequence. Annotated pseudogenes, transposable-element-related and RNA-coding genes were discarded from both genomic data sets. The final data sets comprised upstream sequences of 26,140 genes from Arabidopsis and 49,472 from rice (Fig. 1a). These were first used to generate randomised data sets that served in the following analyses as a background model. The background model was generated by reshuffling the individual upstream sequences while maintaining their original single-nucleotide frequencies. The reshuffling process was repeated 100 times to generate 100 different data sets of randomised 1-kb upstream sequences for Arabidopsis and rice, respectively (Fig. 1a, bottom right).

**Figure 2:**
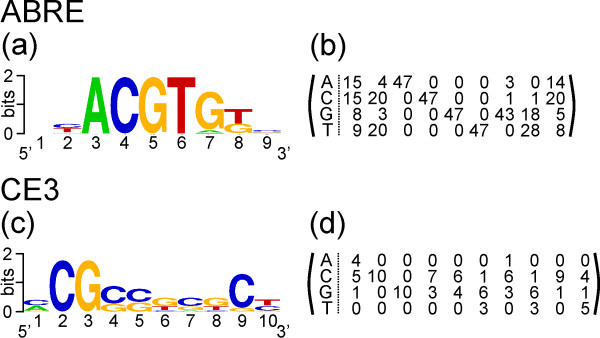
**Sequence logos and matrices for ABRE and CE3 used in this study**. Sequence logos were created online using the Weblogo resource [48]. (a) ABRE sequence logo; (b) ABRE frequency matrix; (c) CE3 sequence logo; (d) CE3 frequency matrix.

ABREs and CE3s were then located in the 1-kb upstream sequences applying a matrix-based *in-silico *screening procedure (Fig. 1b). Element-specific frequency matrices were generated from upstream sequences known to confer ABA responsiveness. The sequence logos as well as the frequency matrices for ABRE and CE3 are shown in Fig. 2. Candidate sequences for ABREs or CE3s were considered as positive hits if the score, i.e. the sum of scores at each position of the matrix, was ≥ 95% of the maximally attainable score (Fig. 1b). Application of this cut-off led to the identification of 28 putative ABREs and ten different CE3s (Additional files 1 and 2). The distance to the ATG was calculated for each detected ABRE and CE3 (Fig. 1c). Distances between pairs were computed whenever two or more non-overlapping elements co-localised within the same 1-kb sequence (Fig. 1d).

### Single ABRE frequency in Arabidopsis promoters is similar to that in randomised sequences

We first used the motif models to identify all single occurrences of ABRE and CE3 in Arabidopsis and rice promoters. The significance of the results was assessed by comparing the number of occurrences of each motif in the genomic data set with the number of occurrences in the randomised data sets (which represents the value expected in case the motif occurred by chance). The relevance of strand orientation was assessed by counting the number of matches on either strand. For comparison purposes between both species, the number of occurrences was normalized to the number of motifs per 10,000 genes (Table 1).

**Table 1:** Genome-wide occurrence of ABRE and CE3 in Arabidopsis and rice.

CRE	*Species*	No. of elements		CREs per 10,000 genes	
			
		Genomic data set	Randomised data set	Genomic data set	Randomised data set
ABRE	*A. thaliana*	3,829	3,789 ± 75	1,465	1,450 ± 29
	*O. sativa*	11,282*	8,819 ± 98	2,280*	1,783 ± 20
CE3	*A. thaliana*	52	60 ± 8	20	23 ± 3
	*O. sativa*	4,126*	755 ± 31	787*	165 ± 5

*Significantly different from the background model at P < 0.01 (z-test).

Our analysis revealed that in both species ABRE shows no orientation preferences. However, we found that the number of ABREs per 10,000 genes is about 1.5 times higher in rice than in Arabidopsis (2,280 vs. 1,465). Interestingly the number of ABREs found in the randomised data sets of Arabidopsis was almost identical to that in the genomic data set (3,789 ± 75 vs. 3,829; cf. Table 1). In contrast, in rice there were significantly less (about 25%) ABREs in the randomised than in the genomic data sets (8,819 ± 98 vs. 11,282; cf. Table 1). Thus, the relative abundance of ABREs was higher in rice than in Arabidopsis.

**Figure 3:**
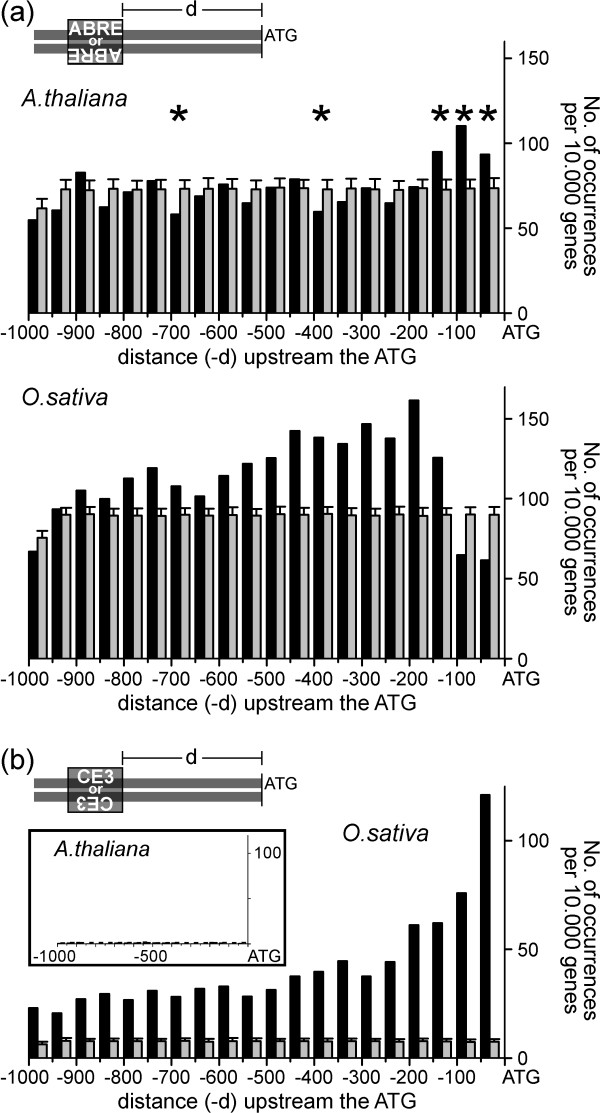
**Distribution of ABREs and CE3s upstream of peptide-coding genes in *A. thaliana *and *O. sativa***. Data are summarized as histograms with a bin size of 50 bases. For inter-specific comparison purposes, results are displayed as occurrences per 10,000 genes. The grey bars represent the values obtained for the background model (mean ± SD). (a) ABREs on both the [+] or the [-] strand, in *A. thaliana *(upper panel), and *O. sativa *(lower panel). The asterisks in the upper panel indicate values significantly different from the background model. In the lower panel all values, except for those at 800–850 bases and 900–950 bases, were significantly different from the background model; (b) CE3s in *O. sativa *and *A. thaliana *(inset).

In Arabidopsis, the distance of ABREs to the translational start codon (ATG) differed only slightly from that of a random distribution while the number of elements was significantly higher only at short distances from the ATG (0–150 bp). In contrast, the number of occurrences in the intervals 351–400 bp and 651–700 bp was significantly smaller than in the randomised data sets (Fig. 3a, top panel). As expected for a suitable background model, ABREs in the reshuffled data sets were evenly distributed (Fig. 3a, grey bars). In rice the distribution plot for ABRE shows marked differences between the randomised and the genomic data sets. For nearly all distance intervals, the number of ABREs was significantly higher in the genomic data set (Fig. 3a, bottom panel). Surprisingly, ABREs in rice showed a positional preference different from that in Arabidopsis. Whereas in the latter ABREs tend to be located close to the ATG, in rice they were underrepresented in that region.

### CE3 is overrepresented in rice but almost absent in Arabidopsis

The CE3 screen of the Arabidopsis genome revealed only 52 matches among the 26,140 genes analysed. This small number corresponds to 20 elements per 10,000 genes, which is not significantly different from the number obtained for the corresponding randomised data sets (60 ± 8 in the entire set, or 23 ± 3 CE3 elements per 10,000 genes). In rice CE3 was about 80 times more frequent than in Arabidopsis (4,126 occurrences in the complete genome, or 787 per 10,000 genes) and at least five times more abundant than in the corresponding randomised sequences (165 ± 5 per 10,000 genes) (cf. Table 1). As with ABRE the number of CE3s found on either DNA strand was almost identical.

The distribution of distances of CE3s relative to the ATG is shown in Figure 3b. In the genomic data set most CREs are located close to the ATG (within 200 bp). The inlay panel shows the distribution of distances of CE3s found in Arabidopsis, using the same scale as in rice. From this representation it becomes evident that CE3s are abundant in rice but almost absent in Arabidopsis.

Additional files 3 and 4 contain a list of all Arabidopsis and rice genes harbouring a single ABRE or CE3 in their upstream sequences. The distances of the CREs to the translation start codon (ATG) as well as their strand orientations are indicated. The tables additionally include (i) a description of the protein encoded by the gene's ORF; (ii) the length of the 5'UTR of the gene (where known); and (iii) the location of the element with respect to the 5'UTR. Information regarding the 5'UTR is available for around 50 percent of all genes. Nevertheless, our analysis showed that in Arabidopsis and rice CREs are in most cases located upstream of the 5'UTR.

### ABRE-ABRE pairs are overrepresented in Arabidopsis and rice

It has been shown in Arabidopsis, rice, barley and other plant species that ABA responsiveness relies on the interaction of two CREs in the promoter region [14,16,20,21]. ABRE has been shown to form functional ABA-responsive complexes together with CE3 [14,16,20,21], but also a pair of ABREs can form such a complex [16,20,28]. Therefore, we investigated the occurrence of all possible pairs (ABRE-ABRE, CE3-CE3, ABRE-CE3, and CE3-ABRE) in both plant species. In doing so, we considered all possible strand orientations, distances between pairs, and distances of pairs to the ATG (Fig. 1d). In the following, the first element of a given pair is the one more distant to the ATG (distal element).

The number of ABRE-ABRE pairs detected in the genomic and randomised data sets is shown in Table 2. For both species we found more ABRE-ABRE pairs in the genomic data sets than in the randomised data sets. Specific structural patterns, not detected when looking at single ABREs, became evident when looking at ABRE-ABRE pairs, especially in Arabidopsis. The ratio between genomic and randomised data was about unity for single ABREs in Arabidopsis, while the ratio for ABRE-ABRE pairs was about 2.6. Both these numbers were higher in rice, with single ABREs being 1.3 times more abundant than in the randomised data sets, and ABRE-ABRE pairs 3.5 times, respectively. Hence, in both species the pairing process increased the relative difference between genomic and randomised data by a factor of approximately 2.6.

**Table 2:** Genome-wide distribution of ABA-responsive element pairs

*CRE pair*	*Species*	No. of pairs		Pairs per 10,000 genes	
			
		Genomic data set	Randomised data set	Genomic data set	Randomised data set
*ABRE-ABRE*	*A. thaliana*	735	278 ± 23	287	108 ± 9
	*O. sativa*	2,759	783 ± 36	558	158 ± 7
CE3-CE3	*A. thaliana*	0	1 ± 0.5	0	0 ± 0
	*O. sativa*	713	22 ± 6	144	5 ± 1
ABRE-CE3	*A. thaliana*	10	5 ± 2	4	2 ± 1
	*O. sativa*	912	70 ± 9	184	14 ± 2
CE3-ABRE	*A. thaliana*	13	5 ± 2	5	2 ± 1
	*O. sativa*	335	72 ± 9	68	15 ± 2

Results are significantly different from the background model at P < 0.01 (except for n = 0) (z-test).

ABRE-ABRE pairs showed no strand preference in either genomic data set (data not shown). However, we found that most pairs were located in relatively close proximity to the ATG. The mean distance from the proximal end of the complex to the ATG was similar for both species (354 and 390 bp for Arabidopsis and rice, respectively; Table 3). Furthermore, in both cases around half the pairs showed a maximal distance to the ATG of 350 bp (not shown).

**Table 3:** Structural properties of ABRE and CE3 pairs

CRE pair	Species	Mean gap length (bp)	Mean distance to the ATG (bp)
*ABRE-ABRE*	*A. thaliana*	236	354
	*O. sativa*	186	390
CE3-CE3	*A. thaliana*	n.a.	n.a.
	*O. sativa*	157	326
ABRE-CE3	*A. thaliana*	n.a.	n.a.
	*O. sativa*	340	245
CE3-ABRE	*A. thaliana*	n.a.	n.a.
	*O. sativa*	313	386

n.a., not applicable because of absence or low abundance.

**Figure 4:**
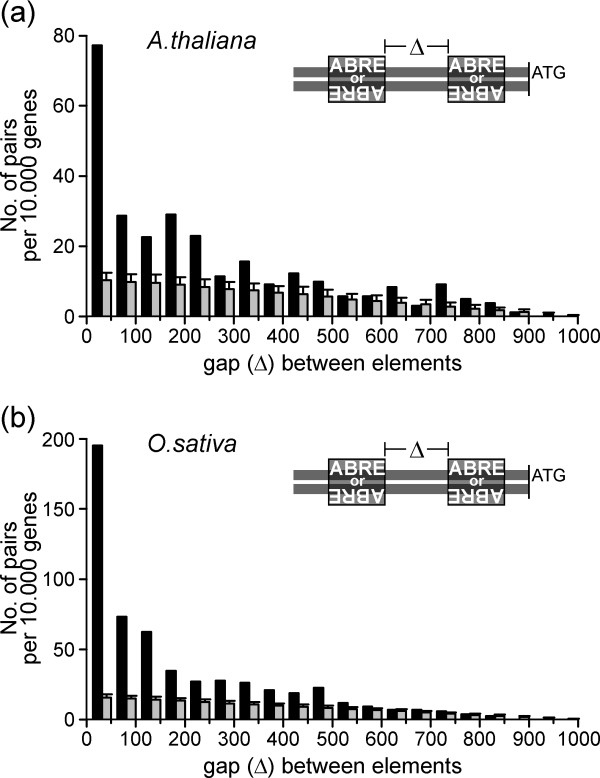
**Gap length distribution of ABRE-ABRE pairs**. (a) *A. thaliana *or (b) *O. sativa*. Data are presented as in Figure 3.

Despite the close proximity of many complexes to the ATG, the CREs were only rarely located within the 5'UTR (13% in Arabidopsis and 5% in rice, respectively; see Additional files 5 and 6). In both species the gap between ABRE-ABRE pairs was typically small (Fig. 4). The mean gap length was 236 bp for Arabidopsis and 186 for rice (Table 3). For more than half the pairs found in both genomes the spacing was ≤ 200 bp. Taken together, in both genomes ABRE-ABRE pairs exhibit specific structural preferences with respect to abundance, gap length and distance to the ATG.

### CE3-CE3 pairs are absent in Arabidopsis but exhibit preferred orientation in rice

**Figure 5:**
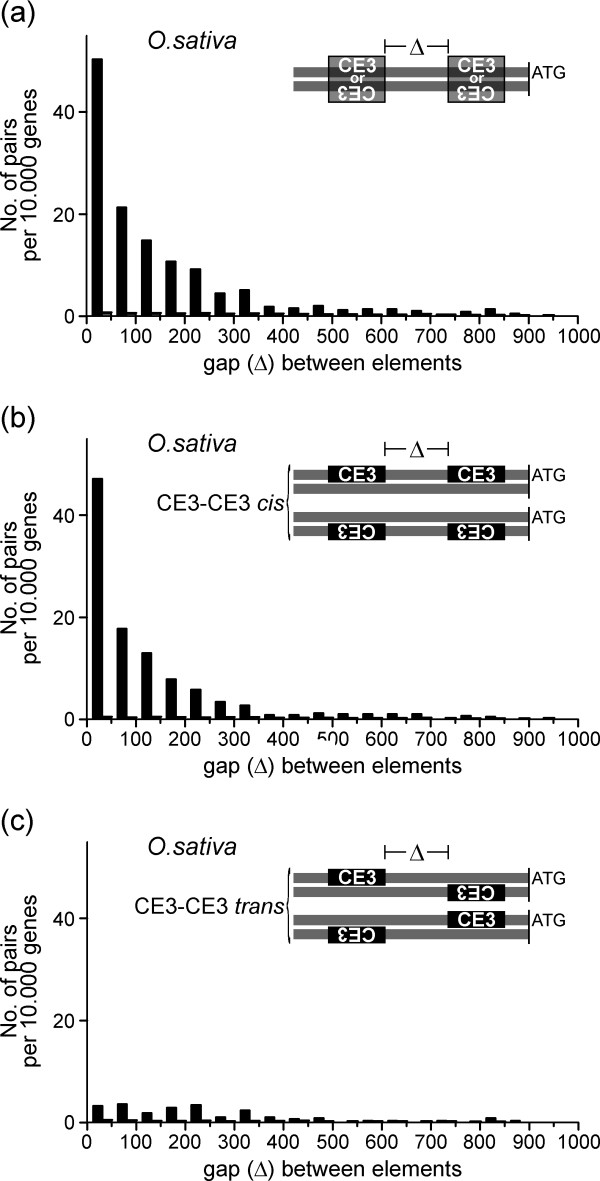
**Gap length and orientation preferences of CE3-CE3 pairs**. Distribution of gap lengths (a) for all CE3-CE3 pairs irrespective of their orientation; (b) for *cis*-oriented pairs; and (c) for *trans*-oriented pairs. Data are presented as in Figure 3.

As indicated above, CE3 is almost absent in Arabidopsis, and without exception the few hits obtained represented single matches, i.e. no CE3-CE3 pairs were found. In rice, on the other hand, the number of CE3-CE3 pairs observed in the genomic data set was almost 30 times larger than in the randomised data sets (Table 2), and the complexes were located close to the ATG, with a mean distance of 326 bp (Table 3). The gap size between CREs was typically small, with a mean of 157 bp, and more than 80% of the pairs had gap lengths ≤ 300 bp (Fig. 5a). Remarkably, and in contrast to ABRE-ABRE pairs, which do not exhibit orientation preferences, CE3-CE3 pairs displayed a clear preference for the *cis *orientation [+/+] or [-/-], rather than for the *trans *orientation [+/-] or [-/+] (Fig. 5b, c). In sum, CE3-CE3 pairs displayed specific structural preferences with respect to abundance, gap length, distance to the ATG, and mutual orientation.

### ABRE and CE3 pairs exhibit positional preferences in rice

**Figure 6:**
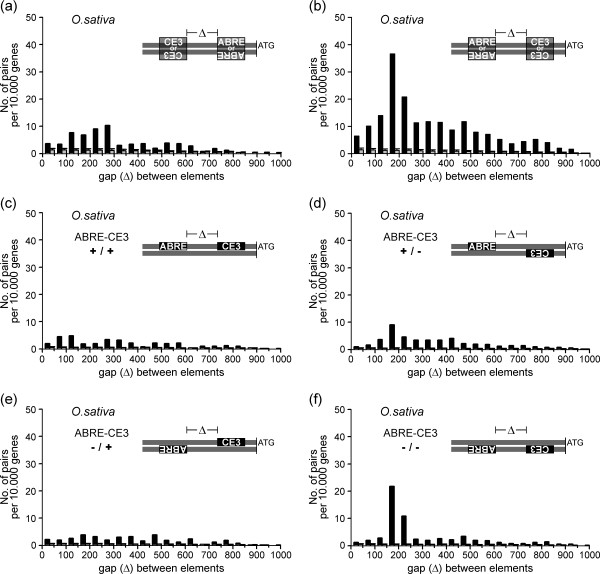
**Abundance, distance and orientation preferences of ABRE-CE3 and CE3-ABRE pairs in *O. sativa***. Orientation-independent gap length distribution for (a) CE3-ABRE pairs, and (b) ABRE-CE3 pairs. Orientation-dependent gap length distribution for ABRE-CE3 pairs (c) [+/+]; (d) [+/-]; (e) [-/+]; (f) and [-/-]. Data are presented as in Figure 3.

As expected from the low abundance of CE3s in Arabidopsis, ABRE-CE3 pairs were almost absent in this species. Only ten ABRE-CE3 and 13 CE3-ABRE pairs were found in the complete genomic data set. Because of their small number, no further analyses were performed. In contrast, in rice both pairs, ABRE-CE3 and CE3-ABRE, were nearly ten and five times more abundant than in the randomised data sets, respectively (Table 2). Interestingly, ABRE-CE3 pairs were almost three times more frequent than CE3-ABRE pairs, pointing to a non-arbitrary positioning of these elements. Further striking differences became evident after analysing the gaps within pairs. Whereas CE3-ABRE pairs exhibited a smooth distance distribution, with most pairs showing a gap length of 151 to 300 bp (Fig. 6a), ABRE-CE3 pairs showed a large peak at a gap length of 151–200 bp (Fig. 6b). Despite the different distribution patterns, the mean gap lengths were similar: 340 bp for ABRE-CE3 and 313 bp for CE3-ABRE (Table 3). From all four pairs analysed in this study, ABRE-CE3 showed the shortest mean distance to the ATG (245 bp), while CE3-ABRE showed a mean distance of 386 bp, which was similar to the mean distance for ABRE-ABRE pairs (390 bp; Table 3).

For both ABRE-CE3 and CE3-ABRE pairs, we did not observe a preference for *cis *or *trans *orientation (not shown). However, for both pairs the ATG-proximal CRE was preferably located on the minus-strand. Two-thirds of CE3-ABRE pairs were found in the [+/-] or [-/-] orientation (not shown). Similar results were obtained in the case of ABRE-CE3 pairs. The orientations [+/-] (Fig. 6d) and especially [-/-] (Fig. 6f) were more prevalent than the [+/+] and [-/+] orientations (Fig. 6c, e). Summarising, the analysis of CE3-ABRE and ABRE-CE3 pairs in rice revealed inherent functional constraints affecting their position and orientation. Interestingly, ABREs were not only more frequent in the distal position, but they were also subject to specific constraints with respect to their abundance, gap length, distance between the elements and the ATG, and orientation of the proximal CRE relative to the ATG.

### Biological relevance

To better understand the biological implications of our observations, the putative ABA-responsive genes identified in our screen were compared with genes regulated by ABA, and which had been identified in the course of expression profiling experiments [29-31]. The results presented in Table 4 show the number of Arabidopsis genes predicted by our screening approach and those that were experimentally verified. The number of ABA-responsive genes confirmed experimentally increased from 15 to 20 percent when ABRE-ABRE pairs rather than single ABREs were considered. Thus the pairing process results not only in a better distinction between the genomic data set and the background model (see above), but also allows a more accurate identification of functional ABRCs in plant genomes.

**Table 4:** Genes identified *in silico *and verified experimentally in *A. thaliana*

CRE	*In-silico *prediction	Verified [29, 30]	%
ABRE	2,669	404	15.1
ABRE-ABRE	331	68	20.5

In rice, the limited number of publicly available expression profiling experiments revealed only 226 ABA-responsive genes (0.46% of the genome) [32,33]. Despite the small number of ABA-responsive genes confirmed experimentally, the overlap between genes predicted by our computational screening and those experimentally verified also increased when at least one CRE pair, rather than a single CRE, had been identified in the upstream region (data not shown). However, the significance of these findings will only be confirmed once more expression data become available.

Among the rice genes identified in our study as harbouring at least one CRE pair (ABRE-ABRE, ABRE-CE3, CE3-ABRE, or CE3-CE3), there are some that are already being employed experimentally as ABA-responsive markers, such as: two protein phosphatase 2C genes (LOC_Os03g16170.1 and LOC_Os09g15670.1); the *HVA22 *gene (LOC_Os08g36440.2); the *RAB 16D *gene (LOC_Os11g26750.1); and genes encoding LEA (late-embryogenesis-abundant) proteins [34].

Strikingly, the largest number of CRE pairs (nine) was found in the promoter region of a putative NAC transcription factor gene (LOC_Os01g66120.2). Using the recently established Plant Transcription Factor Database (PlnTFDB) [35] we were able to identify putative LOC_Os01g66120 orthologous genes in both Arabidopsis (AGI code At1g01720; gene *ATAF1*) and *Populus trichocarpa *(grail3.0003068301; protein ID:643591). The *ATAF1 *promoter harbours two ABREs, and a single ABRE is present in the upstream region of the *P. trichocarpa *gene. ATAF1 has been shown to function as a transcription factor in an ABA-dependent abiotic stress response pathway [36]. Our findings support an evolutionary conservation of this regulatory network in monocots and dicots, and stress the importance of NAC transcription factors in ABA-dependent signalling pathways beyond their role as ABA-independent *trans*-activators [11].

## Discussion

Regulation of gene expression is one of the chief mechanisms of phytohormone action. The identification of genes responsive to external or internal stimuli often relies on experimental data derived from mutant or knockout plants, and more recently also expression profiling experiments [19,29,30,37,38]. With the availability of the fully sequenced Arabidopsis and rice genomes [39,40], computational methods can additionally be applied to determine CREs in promoter regions of genes expressed differentially upon the action of diverse stimuli, but also to make genome-wide predictions about the expression patterns of genes not yet characterised in detail. In this study we conducted a genome-wide search for CREs known to be involved in ABA-dependent regulation of gene expression, in Arabidopsis and rice. We focussed on ABRE and CE3 as model CREs, and uncovered a number of noteworthy features regarding DNA strand preferences, distances, and orientation patterns inherent to pairs formed by these CREs. These findings will help in genome-wide scans for genes sharing common phytohormone-responsive mechanisms.

The predominantly short gap distances within CRE pairs found in our study suggest that there is a high potential for physical interaction between the transcription factors binding to the CREs involved, as would be expected for ABRCs affecting RNA polymerase II activity.

One of the most remarkable findings of our study was the virtual absence of CE3 in upstream sequences of *Arabidopsis thaliana*. This finding might explain why despite numerous reports of CE3 sequences in a number of monocot plant species (barley, maize, rice) [15,18,20,41,42] so far no functional CE3 has been reported in Arabidopsis or other dicots. To our knowledge, only Suzuki and colleagues have reported CE3-like sequences in association with ABRE in Arabidopsis upstream sequences, albeit the association was not statistically significant [43]. The marked prevalence of ABRE-ABRE pairs in Arabidopsis is in agreement with other reports indicating that they are probably the most common receptor-binding complexes in this species [27]. We have further indications that DRE-ABRE and MYB-ABRE pairs are significantly over-represented in upstream sequences of Arabidopsis [44].

Considering the importance of CEs in specifying ABA responsiveness in combination with ABRE [15,18], it is tempting to hypothesise that there has been a gain or a loss and subsequent diversification of CE3 usage along the evolutionary path. The fact that CE3 is virtually absent in Arabidopsis points to an evolutionary deep branching point. Analysis of upstream sequences from other plant species will be needed to reveal how the most recent common ancestor of dicots and monocots looked like and whether the virtual absence of CE3 is common among dicots.

Using a combined approach to define ABRE-CE3 pairs in rice (ABRE matrix and CE3 consensus sequence) and by constraining the distance between elements to maximally 20 bp, Ross and Shen recognized – as we do in our study – that CE3 elements show a preference for the minus strand [45]. From these observations, we infer that the orientation bias may reflect the evolutionary history of these elements, since in rice these elements are functional in either orientation [18]. However, differences in orientation may perhaps lead to differential transcription efficiencies.

By investigating all kinds of arrangements between pairs of ABREs and CE3s, taking all distances between CREs into account without any restrictions, and by using a more complex definition of CE3, our screening approach complements and enhances the computational approach applied by Ross and Shen. One of our findings was that distal ABREs were three times more frequent than proximal ABREs in ABRE-CE3 pairs (cf. Table 2). Concurrently there was a clear bias towards CE3 elements in the reverse orientation, thus forming [+/-] or [-/-] pairs in combination with ABRE, or towards CE3s on the same strand in the case of CE3-CE3 pairs. Since only few studies have dealt with the biological relevance of such spatial arrangements, we would like to draw the attention to these salient genomic features.

Thus far the combinatorial potential of CREs has been mostly understood in terms of motif co-occurrence. However, Nguyen and D'Haeseleer [46], working on yeast, showed to which extent nature may exploit spatial arrangement as another dimension of combinatorial power in transcriptional regulation. The precise positioning of CREs within a given promoter might thus directly modulate gene expression by affecting the three-dimensional structure of the transcriptional machinery. This adds another level of complexity, applied to the fine-tuning of transcriptional regulation. Features such as orientation and order of the elements in a promoter underline regulatory mechanisms which go beyond the mere presence, absence or spacing between motifs or distance to the transcriptional start site.

The results of the *in-silico *genome-wide analysis presented here provide additional clues about the relevance of orientation and combination of motifs in ABA-responsive genes, now open for validation by targeted gene expression analyses. The fact that ABA responsiveness of only few of the *in-silico*-predicted genes has been experimentally verified probably reflects technical limitations that hinder detailed gene expression studies, e.g. in highly specialised (and thus, often rare) cell types. A further complication stems from our incipient knowledge about the number of ABA-responsive genes in different tissues and developmental stages, and the responsible CREs and their structural arrangements. ABA-dependent processes highly confined in space and time are technically difficult to unravel, but once we have built a robust understanding of the interplay of CREs and transcription factors, we will be in a position to make accurate predictions and design appropriate experiments to confirm those predictions.

## Conclusion

In the present study we have used a matrix-based search algorithm to show that the ABA-responsive elements ABRE and CE3 are not equally common in plant genomes of different species. ABRE-ABRE pairs form ABA-responsive complexes that are functional and relevant in Arabidopsis and rice. The low abundance of CE3s in Arabidopsis, as opposed to its experimentally verified presence in rice, barley and maize, suggests a preferential role for CE3 in ABA-dependent gene regulation in monocotyledonous species. In rice, the orientation preferences of CE3 further suggest that this element might be typical of monocots, and that its orientation bias may serve to modulate ABA responsiveness of genes and their transcriptional activation potential.

## Methods

### Upstream sequences

The Arabidopsis genomic sequences were downloaded from TIGR (release 5.0, January 2004) [26]. Gene coordinates were used to map the translation start codon (ATG) and to extract the upstream sequences. In total 29,845 1-kb sequences upstream of the ATG were automatically extracted. All upstream sequences that correspond to genes that do not code for proteins or oligopeptides were discarded (3,705 sequences). The final database of *A. thaliana *1-kb upstream sequences consisted of 26,140 individual sequences.

A database of 1-kb upstream sequences from *Oryza sativa *was downloaded from TIGR (release 4.0, January 2006) [26]. Upstream sequences were cleared of transposable elements and related gene models using the gene identifiers of transposable-element-related gene models provided by TIGR (13,355 gene identifiers) and applying the EMBOSS program "seqret" [47]. The file used for the screening consisted of 49,472 sequences.

### Randomised sequences

Randomised sequences were generated by shuffling the extracted upstream sequences using the program "shuffleseq" from EMBOSS [47]. During the shuffling procedure, the single-nucleotide frequency of each sequence is maintained, but the nucleotide order is randomly shuffled.

### CRE models

Frequency matrices were constructed utilising a set of ABREs and CE3s obtained from the literature. The set of 47 sequences containing an ABRE site included genes from *Arabidopsis thaliana *[19,28], barley [18], maize [41], rice [20], and cotton [17]. Ten CE3 sequences were obtained from barley [15,18], maize [41], and rice [20,42]. The sequences were manually aligned, and the position-specific frequency matrices were calculated by counting the occurrence of every nucleotide at each position. The sequence logos and the corresponding matrices are presented in Figure 2.

### Searching single binding sites

The genomic and randomised data sets were screened for single occurrences of the CREs ABRE and CE3 on the plus and minus strands, using the frequency matrices. The program "profit" from EMBOSS [47] was used for screening. For each screened data set the program generates a list of matches, including the sequence identifier, element, score and position for every match. The score is calculated from the frequency matrix, and is the sum of scores at each position of the matrix, expressed as a percentage. An arbitrary 95% cut-off was used. Using this cut-off, 28 ABREs and ten CE3s were obtained (for details about oligonucleotides and scores see Additional files 1 and 2). The position of a match is expressed in terms of the distance to the translation initiation codon (ATG). As depicted in Figure 1c, in case of matches on the plus strand, the distance to the ATG refers to the distance from the last base of the CRE to the translation initiation codon, and for matches on the minus strand, the distance to the ATG refers to the distance from the first base of the CRE to the ATG. Significance analyses were carried out using a z-test. For the calculations, the mean value and the standard deviation obtained from the randomised sequences were used, as well as the occurrence in genomic data sets. A significance threshold of P = 0.01 was chosen.

### Assessment of CRE pairs

For each data set all ABREs and CE3s found on the plus and minus strands were merged into a single file. For each gene in a file all possible ABRE and CE3 pairs were determined. The distance between pairs of CREs was calculated as depicted in Figure 1d, and only non-overlapping matches were considered for further analysis. The proximal CRE determined the distance of a pair to the ATG.

## Authors' contributions

JLGP generated the randomised datasets and frequency matrices, and performed the computational analyses. DMRP and JLGP wrote the Perl scripts for the analyses of the datasets. ID did the statistical analyses and figure design. All authors contributed to the analytical design and to the drafting of the manuscript. BMR supervised the group at the University of Potsdam. All authors have read and approved the manuscript.

## Supplementary Material

Additional file 1 List of ABREs detected with the matrix shown in Fig. 2b. Click here for file

Additional file 2 List of CE3s detected with the matrix shown in Fig. 2d. Click here for file

Additional file 3 Single occurrences of ABRE in *Arabidopsis thaliana*. Click here for file

Additional file 4 Single occurrences of CREs in *Oryza sativa*. Click here for file

Additional file 5 ABRE-ABRE pairs in *Arabidopsis thaliana*. Click here for file

Additional file 6 Pairs of CREs in *Oryza sativa*. Click here for file
