# Supplementary material for: Genome-wide analysis of ABA-responsive elements ABRE and CE3 reveals divergent patterns in Arabidopsis and rice
Source: BMC Genomics. 2007 Aug 1;8:260. doi: 10.1186/1471-2164-8-260 (PMC2000901; doi:10.1186/1471-2164-8-260)
Supplement: Additional file 1 — List of ABREs detected with the matrix shown in Fig. 2b. [file 1471-2164-8-260-S1.pdf]

Gómez-Porras et al.
Genome-wide analysis of ABA-responsive elements ABRE and CE3 reveals divergent patterns in Arabidopsis and rice

Additional File 1:

List of ABREs detected with the matrix shown in Fig. 2b

Oligonucleotide	Matrix Score	Elements per 10,000 genes	
		A. thaliana	*O. sativa*
ACACGTGTC	100	123	69
ATACGTGTC	100	29	26
CCACGTGTC	100	116	163
CTACGTGTC	100	23	35
ACACGTGTA	98	81	48
ATACGTGTA	98	68	56
CCACGTGTA	98	52	58
CTACGTGTA	98	31	37
TCACGTGTC	98	35	31
TTACGTGTC	98	46	29
GCACGTGTC	97	18	29
GTACGTGTC	97	22	45
ACACGTGGC	96	104	120
ACACGTGTT	96	93	36
ATACGTGGC	96	29	38
ATACGTGTT	96	67	75
CCACGTGGC	96	82	243
CCACGTGTT	96	54	47
CTACGTGGC	96	21	172
CTACGTGTT	96	33	35
TCACGTGTA	96	42	44
TTACGTGTA	96	65	55
ACACGTGTG	95	59	46
ATACGTGTG	95	46	45
CCACGTGTG	95	28	93
CTACGTGTG	95	25	29
GCACGTGTA	95	24	40
GTACGTGTA	95	49	60
