# Supplementary material for: Genome-wide analysis of ABA-responsive elements ABRE and CE3 reveals divergent patterns in Arabidopsis and rice
Source: BMC Genomics. 2007 Aug 1;8:260. doi: 10.1186/1471-2164-8-260 (PMC2000901; doi:10.1186/1471-2164-8-260)
Supplement: Additional file 2 — List of CE3s detected with the matrix shown in Fig. 2d. [file 1471-2164-8-260-S2.pdf]

Gómez-Porras et al.
Genome-wide analysis of ABA-responsive elements ABRE and CE3 reveals divergent patterns in Arabidopsis and rice.

Additional File 2:

List of CEs detected with the matrix shown in Fig. 2d

Oligonucleotide	Matrix Score	Elements per 10,000 genes	
		A. thaliana	*O. sativa*
CCGCCGCGCT	100	0	72
ACGCCGCGCT	98	0	19
CCGCCGCGCC	98	2	282
ACGCCGCGCC	97	1	50
CCGCGGCGCT	97	2	30
ACGCGGCGCT	95	2	13
CCGCCGCTCT	95	7	56
CCGCCGGGCT	95	2	16
CCGCCTCGCT	95	3	46
CCGCGGCGCC	95	0	80
